# Association between *5’-UTR* and *3’-UTR* Polymorphisms of the *TYMS* Gene and Breast Cancer in Kurdish Women of Iraq

**DOI:** 10.31557/APJCP.2021.22.5.1557

**Published:** 2021-05

**Authors:** Kazhal Shekh Mohamad

**Affiliations:** *Department of Medical Laboratory Sciences, College of Sciences, University of Raparin, Ranya, Kurdistan Region of Iraq. *

**Keywords:** TYMS, polymorphism, Kurdish, breast cancer

## Abstract

Breast cancer is a common cancer found among women worldwide. Many polymorphisms can play a role in the development of this disease. The study was conducted to evaluate whether 2R/3R and 6bp insertion/deletion polymorphisms of the *TYMS* gene are associated with breast cancer risk in the Kurdish Iraqi population.

**Materials and Methods:**

DNA was extracted from EDTA-treated peripheral blood samples and included 100 patients affected by breast cancer and 100 controls. Genotyping was performed by PCR-electrophoresis.

**Results:**

Our results showed a significant association between 6bp deletion homozygote genotype in breast cancer patients compared to healthy individuals (P = 0.042). No significant differences were observed for other allelic and genotypic frequencies.

**Conclusion:**

This study, performed for the first time on the Kurdish population, highlighted the need for further studies of the *TYMS* gene polymorphisms.

## Introduction

Breast cancer is one of the most common cancers and the second leading cause of death among women across the world. Many factors are associated with increased breast cancer risk in women. These include her age, family history, reproductive and gynaecologic factors, lifestyle factors including alcohol consumption and lack of physical activity, among others. Likewise, genetic alterations induced by endogenous metabolites and exogenous hazards also contribute to the development of breast cancer (Mavaddat et al., 2010). 

Recently, several single nucleotide polymorphisms have been identified as potential aetiological factors for neoplasm, as well as a being a significant factor in therapeutic responses and prognosis (Tan et al., 2008), and many of them have shown an association with susceptibility to breast cancer (Haghi et al., 2015; Sheikh et al., 2015; Haghi et al., 2021). Thymidylate synthetase (TYMS) enzyme, whose gene is located on chromosome 18, is involved in thymidine biosynthesis. In this sense, it has an effective role in DNA replication and cell proliferation (Rode et al., 2015). Increased expression of the *TYMS* gene has been reported in some cancers (Jiang et al., 2019; Cho et al., 2020). There are common polymorphisms in* 5’-UTR*, and *3’-UTR* of *TYMS* gene was reported. In humans, the *TYMS *gene is widely polymorphic, and a variable number of tandem repetitions (VNRT) are shown in the 5’-UTR, in most cases, with two (2R) or three repetitions (3R) of a sequence of 28 bp (rs34743033). Studies have shown that the homozygotes 3R/3R increase expression of TYMS mRNA when compared to cells in 2R/2R homozygotes (Kawakami et al., 1999). Another *TYMS* polymorphism, a 6-bp deletion/insertion (TAAAGT) in the 3’-UTR (rs16430), has also been reported (Pastorakova et al., 2017; Marsh, 2005). Polymorphisms in the *TYMS* gene can affect the expression and stability of mRNA, and therefore affect protein expression. Therefore, it seems that polymorphisms that affect the rate of expression can increase the predisposition to cancer (Abbasian et al., 2020). The impact of single nucleotide polymorphisms (SNPs) in the TYMS enzyme in the risk of breast cancer has been investigated among most ethnic background populations in the world, but not among women of Kurdish Iraqi populations. Kurds, one of the most ancient indigenous people of the Mesopotamian plains, are currently distributed in Turkey, Iran, Iraq and Syria (Gunes, 2019). For the ﬁrst time, this study intends to obtain the Kurdish *TYMS* gene polymorphism and to study the relative contribution of its variants to breast cancer risk in the Iraqi Kurdish population. 

## Materials and Methods

*Samples*

100 patients with breast cancer who had been admitted to a Rizgary Hospital, Medical Oncology Department (KRG), Iraq, and a 100 randomly selected healthy controls, took part in this case-control study. After gaining informed consent, 2cc of EDTA-whole blood sample was collected and kept at -20°C until DNA extraction. Demographic characteristics and clinical data of patient and control samples were also collected. The study was approved by the scientific committee of Medical Laboratory Science, College of Science University of Raparin. 

*DNA extraction and genotyping*

DNA was extracted using magnetic nanoparticles from the commercial extraction kit manufactured by ZiAViZ Company (www.ziaviz.com). Genotyping of TYMS was then performed by polymerase chain reaction (PCR) with primers flanking the polymorphism of 5’-UTR region, usually presenting a double (2R) and/or triple (3R) tandem repeat, and 3’-UTR +6 bp/+6 bp polymorphism. The sequences of primers used for amplification are shown in Table 1. The final volume of 50 μl of PCR reaction contained 0.2 mM of each dNTP, 2 mM MgCl_2_, 5 μl of 10X PCR Buffer (pH 8.5), 0.1 μM of both primers and 2 Unit Taq polymerase. Eventually 2 μl of template DNA was added per reaction. For PCR cycling, there was an initial denaturation at 95°C for 2 min, followed by a three-step cycle of 20 sec at 95°C, 30 sec at 59°C, and 25 sec at 72°C for 35 cycles, ending with a final extension of 2 min at 72°C. PCR products were analysed by electrophoresis in 15% gel of polyacrylamide and stained with ethidium bromide. 

*Statistical analysis*

In this study, Demographic characteristics expressed as frequency, mean and standard division. Genotype and allele frequencies were compared between case and control groups using the *χ*^2^ test. SPSS version 20 was used for statistical analysis. P value of < 0.05 was considered significant. 

**Table 1:** Primer Sequencing and PCR Product Size of the Studied Polymorphisms

	Primers	PCR product size
5'-UTR	5´- CGTGGCTCCTGCGTTTCC3 -3´	2R allele: 210bp
2R/3R polymorphism	5´- GAGCCGGCCACAGGCAT - 3´	3R allele: 238bp
3'-UTR 6bp Insertion Deletion polymorphism	5´CAAATCTGAGGGAGCTGAGT-3′	Insertion allele: 158bp
	5´CAGATAAGTGGCAGTACAGA-3′	Deletion: 152bp

**Table 2:** Allele, Genotype and Haplotype Distribution of *TYMS *Gene Polymorphisms in Patient and Control Groups

	Patient n=100	Control n=100	Chi square	P-value	OR (95%CI)
rs34743033 (2R/3R)					
2R	101 (50.5%)	91 (45.5%)	0.811	0.368	1.222 (0.825-1.81)
3R	99 (49.5%)	109 (54.5%)			
2R2R	27 (27%)	20 (20%)	1.001	0.317	1.479 (0.765-2.861)
2R3R	47 (47%)	51 (51%)	0.18	0.671	0.852 (0.489-1.484)
3R3R	26 (26%)	29 (29%)	0.1	0.751	0.86 (0.462-1.601)
rs16430 (6bp I/D)					
I	98 (49%)	114 (57 %)			
D	102 (51%)	86 (43%)	2.258	0.133	1.38 (0.93-2.046)
II	27 (27%)	30 (30%)	0.098	0.754	0.863 (0.467-1.596)
ID	44 (44 %)	54 (54%)	1.621	0.203	0.669 (0.383-1.169)
DD	29 (29%)	16 (16%)	4.129	0.042*	2.144 (1.078-4.264)

**Table 3:** Allele and Genotype Distribution of *TYMS* Gene Polymorphisms in Patient and Control Groups

	Patient (n=100)	Control (n=100)	Chi square	P-value	OR (95%CI)
rs34743033 (2R/3R)					
2R	101 (50.5%)	91 (45.5%)	0.811	0.368	1.222 (0.825-1.81)
3R	99 (49.5%)	109 (54.5%)			
2R2R	27 (27%)	20 (20%)	1.001	0.317	1.479 (0.765-2.861)
2R3R	47 (47%)	51 (51%)	0.18	0.671	0.852 (0.489-1.484)
3R3R	26 (26%)	29 (29%)	0.1	0.751	0.86 (0.462-1.601)
rs16430 (6bp I/D)					
I	98 (49%)	114 (57 %)			
D	102 (51%)	86 (43%)	2.258	0.133	1.38 (0.93-2.046)
II	27 (27%)	30 (30%)	0.098	0.754	0.863 (0.467-1.596)
ID	44 (44 %)	54 (54%)	1.621	0.203	0.669 (0.383-1.169)
DD	29 (29%)	16 (16%)	4.129	0.042*	2.144 (1.078-4.264)

I, Insertion allele; D, Deletion allele

## Results

In this case – control study, each group include 100 samples. The mean age for patient group was 46.6 ± 12.6 years, and for control group was 48.5 ± 11.5 years. Demographic characteristics and clinical data of patient and control samples were presented in Table 2. Frequencies are shown at the allelic and genotypic state for the two polymorphisms in regions 5’-UTR and 3’-UTR. Allele 2R is more frequent in patients but is not significantly different (p-value= 0.368). Differences were not significant for 5’-UTR polymorphism genotypes, although the frequency of 2R2R was higher in the patient compared to the healthy sample (p-value= 0.317). In the case of the 3’-UTR polymorphism, there is a slight difference. The frequency of DD genotype shows a significant difference between the two groups of patients and the healthy group (p-value = 0.042) as the DD genotype in patients shows an association with breast cancer. However, at an allelic level, this difference is not significant (p-value= 0.133). Allele and genotype distribution of *TYMS* gene polymorphisms in patient and control groups are summarized in Table 3.

## Discussion

Genetic predispositions and environmental factors are key components in the development of breast cancer. Researchers have shown an association between genetic polymorphisms and susceptibility to a variety of diseases during a human life. TYMS is considered to be one of the important genes that contribute to DNA synthesis, repair mechanisms, metabolism of anticancer drugs and pharmacogenomics (Amstutz et al., 2011; Loganayagam et al., 2013). This study is the first attempt to examine polymorphisms in the *5’-UTR* and *3’-UTR* region of the *TYMS* gene and its association with breast cancer for patients in the Kurdistan Region of Iraq. Kurdish ethnic populations are descended mainly from indigenous inhabitants of Zagros mountains and they currently live in a region surrounded by Iran, Iraq, Turkey and Syria (Gunes, 2019).

Several studies have reported an association between TYMS 5’-UTR and 3’-UTR polymorphisms and breast cancer risk, but contradictory results were obtained in different studies. Although such an association has been reported in some studies, in other populations no association was observed (Justenhoven et al., 2005; Zhai et al., 2006; Graziano et al., 2004; Naushad et al., 2012). These inconsistent results could be due to other genetic factors and the genetic background of different populations.

The results of this study showed that only the DD genotype for 6bp deletion in *3’-UTR* of the *TYMS* gene was significantly more frequent in breast cancer patients than in the healthy controls. The deletion of 6 bp in the 3’-UTR may influence mRNA transcription, stability, and finally, protein levels, and altered enzyme levels could affect breast cancer susceptibility (Guan et al., 2015). As shown in Table 3, except for the DD genotype, no genotype or allele showed a significant difference between the two groups. To develop a comprehensive understanding of the relationship between these polymorphisms and breast cancer, further studies in other populations with a large sample size are recommended. 

This study is important in several respects. First, it examined the association between these polymorphisms and breast cancer in the Kurdish population for the first time. Second, it enabled the molecular detection of these polymorphisms as a pharmacogenetic factor in this population. These polymorphisms in the *5’-UTR* and *3’-UTR *regions of the *TYMS* gene have been shown to influence *TYMS* expression. The *TYMS* gene is an important target for chemotherapy drugs, such as 5-fluorouracil and methotrexate (Marsh et al., 2005). Due to the increase in various studies in the field of cancer treatment in our population (Mjali et al., 2020; Nidhal et al., 2018), genotyping of the polymorphisms is one of the goals of personalised medicine, which will be achieved by studying and developing molecular detection methods.

## Author Contribution Statement

None.

## References

[B1] Abbasian MH, Ansarinejad N, Abbasi B (2020). The Role of Dihydropyrimidine Dehydrogenase and Thymidylate Synthase Polymorphisms in Fluoropyrimidine-Based Cancer Chemotherapy in an Iranian Population. Avicenna J Med Biotechnol.

[B2] Amstutz U, Froehlich TK, Largiadèr CR (2011). Dihydropyrimidine dehydrogenase gene as a major predictor of severe 5-fluorouracil toxicity. Pharmacogenomics.

[B3] Cho YB, Chung HJ, Lee WY (2011). Relationship between TYMS and ERCC1 mRNA expression and in vitro chemosensitivity in colorectal cancer. Anticancer Res.

[B4] Graziano F, Kawakami K, Watanabe G (2004). Association of thymidylate synthase polymorphisms with gastric cancer susceptibility. Int J Cancer.

[B5] Guan X, Liu H, Ju J (2015). Genetic variant rs16430 6bp > 0bp at the microRNA-binding site in TYMS and risk of sporadic breast cancer risk in non-Hispanic white women aged ≤ 55 years. Mol Carcinog.

[B6] Gunes C (2019). The Kurds in a New Middle East: The Changing Geopolitics of a Regional Conflict. Palgrave Macmillan Press.

[B7] Haghi M, Hosseinpour Feizi MA, Sadeghizadeh M, Lotfi AS (2015). 14-bp Insertion/Deletion Polymorphism of the HLA-G gene in Breast Cancer among Women from North Western Iran. Asian Pac J Cancer Prev.

[B8] Haghi M, Ranjbar M, Karari K (2021). Certain haplotypes of the 3’-UTR region of the HLA-G gene are linked to breast cancer. Br J Biomed Sci.

[B9] Jiang H, Li B, Wang F, Ma C, Hao T (2019). Expression of ERCC1 and TYMS in colorectal cancer patients and the predictive value of chemotherapy efficacy. Oncol Lett.

[B10] Justenhoven C, Hamann U, Pierl CB (2005). One-carbon metabolism and breast cancer risk: no association of MTHFR, MTR, and TYMS polymorphisms in the GENICA study from Germany. Cancer Epidemiol Biomarkers Prev.

[B11] Kawakami K, Omura K, Kanehira E, Watanabe Y (1999). Polymorphic tandem repeats in the thymidylate synthase gene is associated with its protein expression in human gastrointestinal cancers. Anticancer Res.

[B12] Marsh S (2005). Thymidylate synthase pharmacogenetics. Invest New Drugs.

[B13] Mavaddat N, Antoniou AC, Easton DF, Garcia-Closas M (2010). Genetic susceptibility to breast cancer. Mol Oncol.

[B14] Naushad SM, Pavani A, Rupasree Y (2012). Association of aberrations in one-carbon metabolism with molecular phenotype and grade of breast cancer. Mol Carcinog.

[B15] Pastorakova A, Chandogova D, Chandoga J (2017). Distribution of the most common polymorphisms in TYMS gene in Slavic population of central Europe. Neoplasma.

[B16] Rode W, Jarmuńa A (2015). Thymidylate synthase-catalyzed reaction mechanism. Postepy Biochem.

[B17] Sheikh A, Hussain SA, Ghori Q (2015). The spectrum of genetic mutations in breast cancer. Asian Pac J Cancer Prev.

[B18] Tan SH, Lee SC, Goh BC, Wong J (2008). Pharmacogenetics in breast cancer therapy. Clin Cancer Res.

[B19] Zhai X, Gao J, Hu Z (2006). Polymorphisms in thymidylate synthase gene and susceptibility to breast cancer in a Chinese population: a case-control analysis. BMC Cancer.

